# Revision total hip arthroplasty using a fluted, tapered, modular stem follow-up method for a mean of three years: A preliminary study

**DOI:** 10.3389/fphys.2023.873584

**Published:** 2023-05-23

**Authors:** Shu-Xing Xing, Qiang Huang, Zheng-Jiang Li, Yong-Kui Li, Zhao-Nan Ban

**Affiliations:** Department of Orthopedic Surgery, Chengdu Fifth People’s Hospital, Chengdu, China

**Keywords:** total hip arthroplasty, revision, modular taper stem, orthopedic, surgery

## Abstract

**Objective:** This study aimed to evaluate the results and complications related to revision total hip arthroplasty within a short-to-medium follow up period.

**Methods:** From January 2016 to January 2020, we reviewed 31 prosthetic hip arthroplasty stem revisions using a fluted, tapered modular stem with distal fixation. The median age of the patients was 74.55–79 years. The survival rate was 100%, and there were no re-revisions. The Harris hip score improved from an average of 36.5 ± 7.8 before surgery to 81.8 ± 6.2 at the final follow-up.

**Results:** The average final follow-up was 36 (24–60) months. During this time, there was no periprosthetic infection, no prosthesis loosening or breakage, and no sciatic nerve injury. Complications included four (12.9%) intraoperative fractures and eight (25.8%) dislocations that had no stem fractures. The postoperative limb was lengthened by 17.8 ± 9.8 mm. In most cases, bone regeneration was an early and important finding. Three cases underwent extended trochanteric osteotomy, and bone healing was achieved by the final follow-up.

**Conclusion:** The modular tapered stem reviewed in this study was very versatile, could be used in most femoral revision cases, and allowed for rapid bone reconstruction. However, a long-term follow-up study is needed to confirm these results.

## Introduction

Total hip arthroplasty (THA) is currently one of the most successful orthopedic procedures being performed in China. With its widespread application, the proportion of revision hip arthroplasty is increasing yearly. Osteolysis, aseptic loosening, periprosthetic fractures, and infection are the main reasons for revision. The purpose of revision THA is to obtain and achieve long-term prosthesis fixation, improve the patients’ life quality, avoid complications, and maintain or restore the host bone of the proximal femur. Studies have shown that in cases of prosthesis loosening with severe bone defects on the femoral side, either a modular or non-modular prosthesis with distal fixation can achieve relatively good outcomes (Huang et al., 2017). Bone defects on the femoral side are always present to some degree during revision and increase the difficulty of the procedure. In particular, when revising a cemented stem, severe bone loss frequently occurs. The removal of the femoral prosthesis, the management of bone defects, deciding between different prostheses, implantation of the new prosthesis, and maintaining the immediate stability of the prosthesis are the essential issues that surgeons must deal with (Chen et al., 2014; Sculco et al., 2015). Cementless total hip revision using a cylindrical, porous-coated, non-modular prosthesis is considered the gold standard in Europe and North America (Paprosky et al., 1999). A non-modular distal-fixed prosthesis has the advantages of simplicity, a competitive price, and reliable fixation; however, the method may present difficulties related to controlling limb length, anteversion, and offset during surgery (Weeden and Paprosky, 2002). In some cases with significant bone loss, intraoperative fixation with cylindrical, non-modular, porous coatings will not be effective (Bingham et al., 2020). These cases reflect similar features, e.g., extensive metaphyseal injury, thinning of the bone cortex, and widening of the femoral cavity, which allows for a smaller than 4-cm scratch fit at the femoral isthmus (Isacson et al., 2000). In these cases, the modular femoral prosthesis enables the surgeon to establish a match between different metaphysis and diaphysis component sizes, select proper offset and anteversion values, and adjust the limb length according to the canal conditions (Böhm and Bischel, 2004).

Several potential concerns have been raised concerning all modular stems, including breakage, corrosion at the taper causing lysis, fracturing at the modular junction, and taper disengagement (Krishnamurthy et al., 1997). A cementless modular prosthesis is the most common hip revision choice worldwide (Moreland and Bernstein, 1995). In 2015, the current authors began using the new Arcos Modular Femoral Revision System (ARCOS) (Zimmer Biomet Inc., Warsaw, Indiana, United States) stems in hip revision procedures. The ARCOS stem offers a wide range of possible combinations for accommodating different variations of anatomy and bone stock. The ARCOS supports proximal fixation and load, which mimics the concept of primary THA with proximal weight-bearing, leading to bone stock preservation and no stress shielding or thigh pain.

The purpose of this study was to evaluate the results following surgery using the ARCOS stem with a focus on clinical outcomes and complications.

## Materials and methods

This retrospective study included 31 patients and reviewed the use of ARCOS stems for revision THA in the Department of Orthopedics of the Chengdu Fifth People’s Hospital, China. The research was approved by the Ethics Committee of the Chengdu Fifth People’s Hospital (no. 2018-009-01) and conformed to the ethical guidelines of the Declaration of Helsinki. All study participants (or their legal guardians) were informed of the risks involved and provided their written consent to be included prior to participating in the study.

All of the patients included in this study were followed up. The participants comprised 18 males and 13 females, aged 55–79 years, with an average age of 74.83 years. The cause of femoral revision was aseptic loosening. The cause of these first replacements included femoral head necrosis in 17 cases, femoral neck fracture in 10 cases, and osteoarthritis secondary to developmental dysplasia of the hip in 4 cases. Preoperative pelvic anteroposterior X-rays, anteroposterior and lateral X-rays of the affected hip, and three-dimensional computed tomography reconstruction scanning (metal suppression) for the hip were conducted, based on the standard parameters.

The initial diagnosis and the reasons for revision are presented in Table 1. Typically, femoral prostheses were cemented stems, and the median age of prostheses was 8 years (0–20 years). All cases were treated using a posterior lateral approach with an average length of 14 (Harris, 1969; Huskisson, 1974; Gruen et al., 1979; Sutherland et al., 1982; Engh and Bobyn, 1988; Engh et al., 1990; Peters et al., 1993; Moreland and Bernstein, 1995; Sivananthan et al., 2017; Picado et al., 2020) cm. The stems were fixed with a steel wire ring, except for three patients, for whom extended trochanteric osteotomy (ETO) was performed. Based on a study conducted by Wagner (Weeden and Paprosky, 2002), the ETO patients had stems fixed with sutures.

**TABLE 1:** General data of the patients included in this study.

Pathology of primary THA	Amount	Paprosky classification of femoral bone defect	Amount
Femoral head necrosis	17	Type 2	5
Femoral neck fracture	10	Type 3A	14
Osteoarthritis secondary to developmental dysplasia of the hip	4	Type 3B	12

“Femoral bone loss” was classified in this study according to Paprosky types. Before surgery, templates were created for the entire study cohort using calibrated X-rays, and the anticipated final results of the surgeries were optimized according to biomechanical parameters, e.g., leg length and offset. The follow-ups took place at the Chengdu Fifth People’s Hospital. The clinical outcomes were assessed at follow-ups using the Harris hip score (HHS) (Engh and Bobyn, 1988; Moreland and Bernstein, 1995; Krishnamurthy et al., 1997). All of the patients, except those undergoing THA due to a periprosthetic fracture, received an HHS preoperatively. Radiographic assessment was conducted postoperatively after 3 months, 1 year, and thereafter at subsequent visits.

### General data

From January 2016 to October 2020, 31 patients with aseptic loosening following a hip replacement underwent THA in our orthopedics department. The ARCOS stems were used on the femoral side for all cases where the femoral bone defect was more severe than a Paprosky type 1.

### Surgical procedure

**FIGURE 1:**
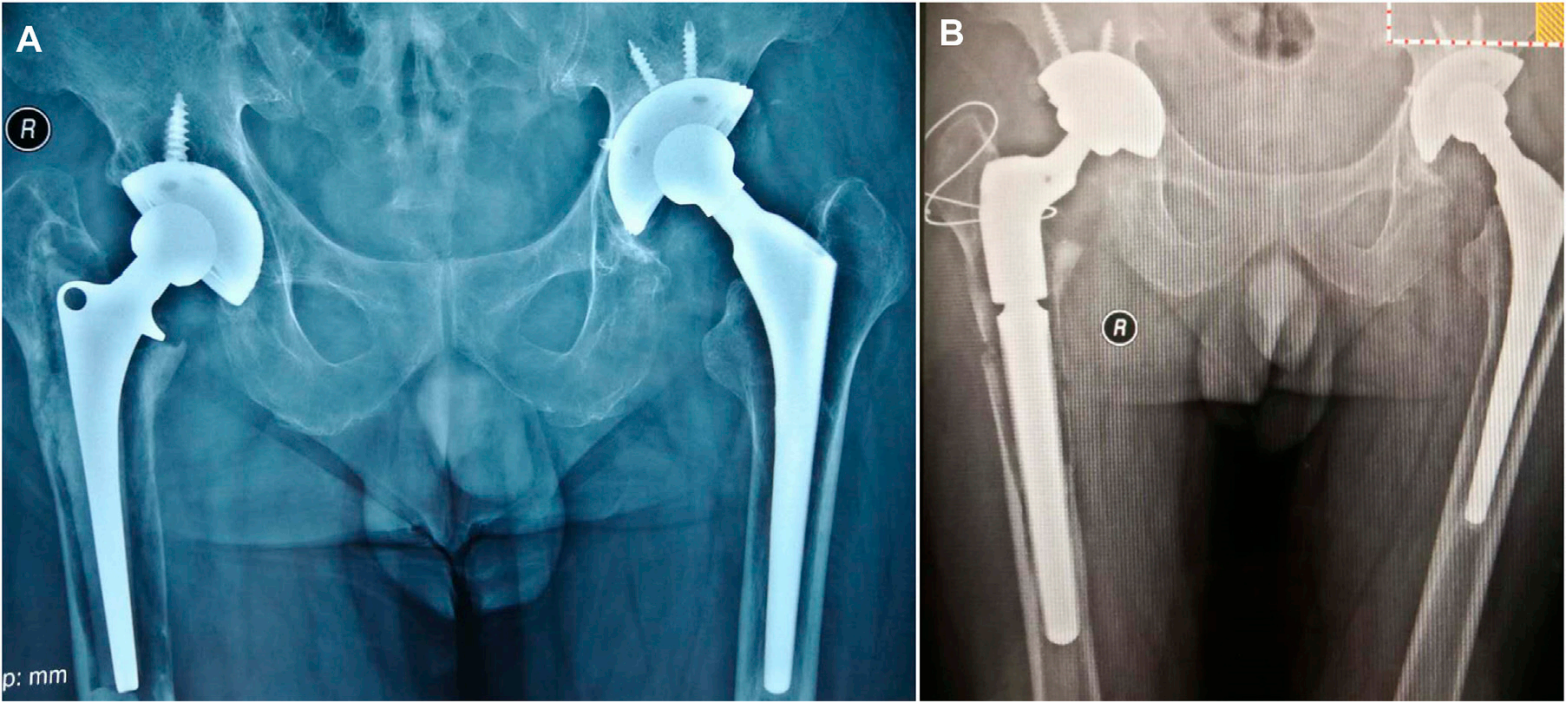
**(A)** A right total hip arthroplasty in a 62-year-old male patient, completed 19 years previously at a different hospital. **(B)** A Paprosky type 3B on the femur side. Bone cement was removed by extended trochanteric osteotomy. An Arcos Modular Femoral Revision System stem was used with impact bone grafting in the canal.

All patients were operated on under general anesthesia. The patient lay in the lateral position and adopted the posterolateral approach. The primary prosthesis was exposed, and the joint fluid and surrounding tissue were sampled for bacterial culture and pathological examination. The femoral prosthesis and acetabular prosthesis were explored to check for the presence of loosening; any loosened prosthesis was removed immediately. For replacement of the acetabular cup via surgery, the original acetabular cup had to be in a severely worn or loosened state. The replacement prosthesis was a cementless cup with a polyethylene liner and a trabecular metal surface. Thus, all patients received a metal-on-poly bearing. If the femoral stem or cement was difficult to remove, ETO was performed to remove the prosthesis (Peters et al., 1993). Scar tissue and residual bone cement in the acetabulum and femur were thoroughly removed. Surgical techniques were meticulously performed to prevent iatrogenic bone loss. A hemispherical cementless cup was tapped in to achieve a press fit. According to the intraoperative condition, impact bone grafting with cancellous bone could be performed. The acetabular cup was fixed with screws. The femoral side was revised with the ARCOS stem. To ensure a stable filling of the isthmus cortex, the distal canal was thoroughly cleaned then slowly reamed in gradual increments until the reamer was completely in contact with the cortical bone. An ETO was applied during surgery, and cerclage cables or allogeneic bone sheet components were inserted, followed by the insertion of the distal implant component. A proximal reamer was used for incremental reaming until the size of the planned proximal was used for fixation (Figure 1). After preparation of the distal femur, the process of implementing the distal prosthesis was begun. The proximal trial prosthesis was inserted and adjusted to the desired anteversion. The femoral head trial was placed, and the hip was reduced to test the hip joint stability and the length of the lower limbs. After a reduction test, the proximal trial was removed and replaced by the proximal component. The selected femoral head was installed and the joint was reduced. The joint cavity was irrigated with a large amount of saline and a drainage tube was positioned before the soft tissue was closed layer by layer.

Postoperative routine infection prevention and anticoagulation measures were taken. Ankle pump exercises and early isometric contraction of the lower limb muscles were ordered to prevent venous thrombosis of the lower extremities. The timing of the drainage tube removal was determined by the drainage volume, and an X-ray was taken between days 3–7 following the surgery. According to the bone defect and the stability of the prosthesis, weight-bearing and walking exercises were individually prescribed. From 3 to 6 months after the surgery, patients were expected to be able to walk with a walker or on two crutches, according to their specific situation.

### Clinical and imaging measurements

After surgery, patients were followed up clinically at 1, 3, 6, and 12 months and then yearly (Harris, 1969). The visual analog score (VAS) was used to assess lower limb pain, and any difference in lower limb length was recorded (Huskisson, 1974).

The X-ray films that were obtained at each follow-up included images for standing anteroposterior pelvis and anteroposterior and lateral hip. In the prosthesis area, radiolucency was classified according to the criteria established by Gruen et al. (1979). The stress shielding was assessed using improved Engh and Bobyn grading methods, which were categorized as light, medium, and heavy degrees (Engh and Bobyn, 1988). The measurement of prosthetic subsidence was the difference between the shoulder of the prosthetic handle and the shoulder of the greater trochanter as indicated on X-ray films after the surgery and during follow up (Sutherland et al., 1982). The femoral stem was classified into three types, based on Engh’s method, i.e., bone ingrowth fixation, stable fibrous fixation, and instability (Engh et al., 1990).

## Results

### Intraoperative complications

**FIGURE 2:**
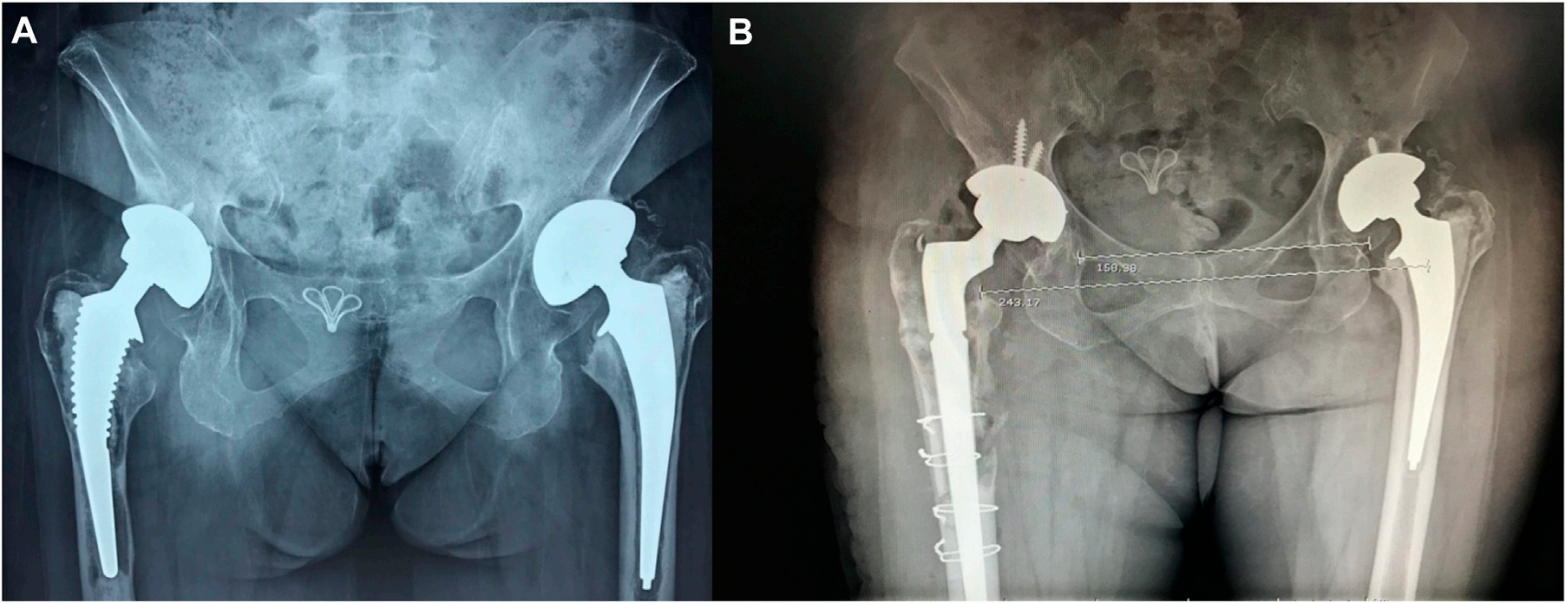
**(A)** A total hip arthroplasty on both sides in a 68-year-old female patient, completed 10 years previously. The acetabulum cup was fixed with a cementless method. The femoral stem was fixed with bone cement. The right hip was painful for 18 months and the left hip for 10 months. **(B)** Paprosky classification of the right side as being type 3B. Intraoperatively, the femoral cortex was fenestrated to remove the bone cement and an Arcos Modular Femoral Revision System prosthesis was implanted. The bone sheet was fixed with wiring.

Femoral fractures occurred in four cases. In the first case, the fracture was located in the proximal and medial femur, and in the second case, the fracture occurred at the bottom of the greater trochanter and was found on a postoperative X-ray film (Figure 2). In the third case, osteoporosis had caused the bone cortex to become thinner, and there was a long fracture line. These three cases were fixed after reduction using a cortical bone plate and steel wire. In the fourth case, a fracture was found during surgery. The X-ray showed that the fracture extended to the supracondylar area; it was fixed using a steel plate.

### Postoperative complications

Dislocation caused by improper posture occurred in eight cases (25.8%). After manual reduction and fixation with a brace, no further dislocations were observed. Up to the final follow up, no periprosthetic fracture occurred. Consequently, no infection of periprosthetic fractures was observed. Low molecular-weight heparin combined with a lower extremity inflatable pump was used to prevent venous thrombosis. No lower extremity deep vein thrombosis and pulmonary embolism were observed after the surgery.

### Radiographic and clinical follow-up

The average follow-up was 36 (24–60) months. The HHS improved from an average of 36.5 ± 7.8 preoperatively to 81.8 ± 6.2 at the final follow-up. The VAS for assessing pain levels decreased from 7.1 ± 2.8 preoperatively to 3.2 ± 1.9 at the final follow-up. Preoperatively, the involved side was 11.3–41.1 mm shorter than the healthy side, with an average shortening of 20.34 ± 8.9 mm. The limb showed a lengthening of 17.8 ± 9.8 mm at the final follow-up. (Table 2).

**TABLE 2:** Comparison of preoperative and the final follow-up radiographic and clinical results.

Index	Preoperatively	Final follow-up	t	P
HHS	36.5 ± 7.8	81.8 ± 6.2	23.58	0.00
VAS	7.1 ± 2.8	3.2 ± 1.9	10.00	0.00
Length of affected limb	20.34 ± 8.9	17.8 ± 9.8	1.62	0.16

Note: HHS: harris hip score; VAS: visual analogue scale.

**FIGURE 3:**
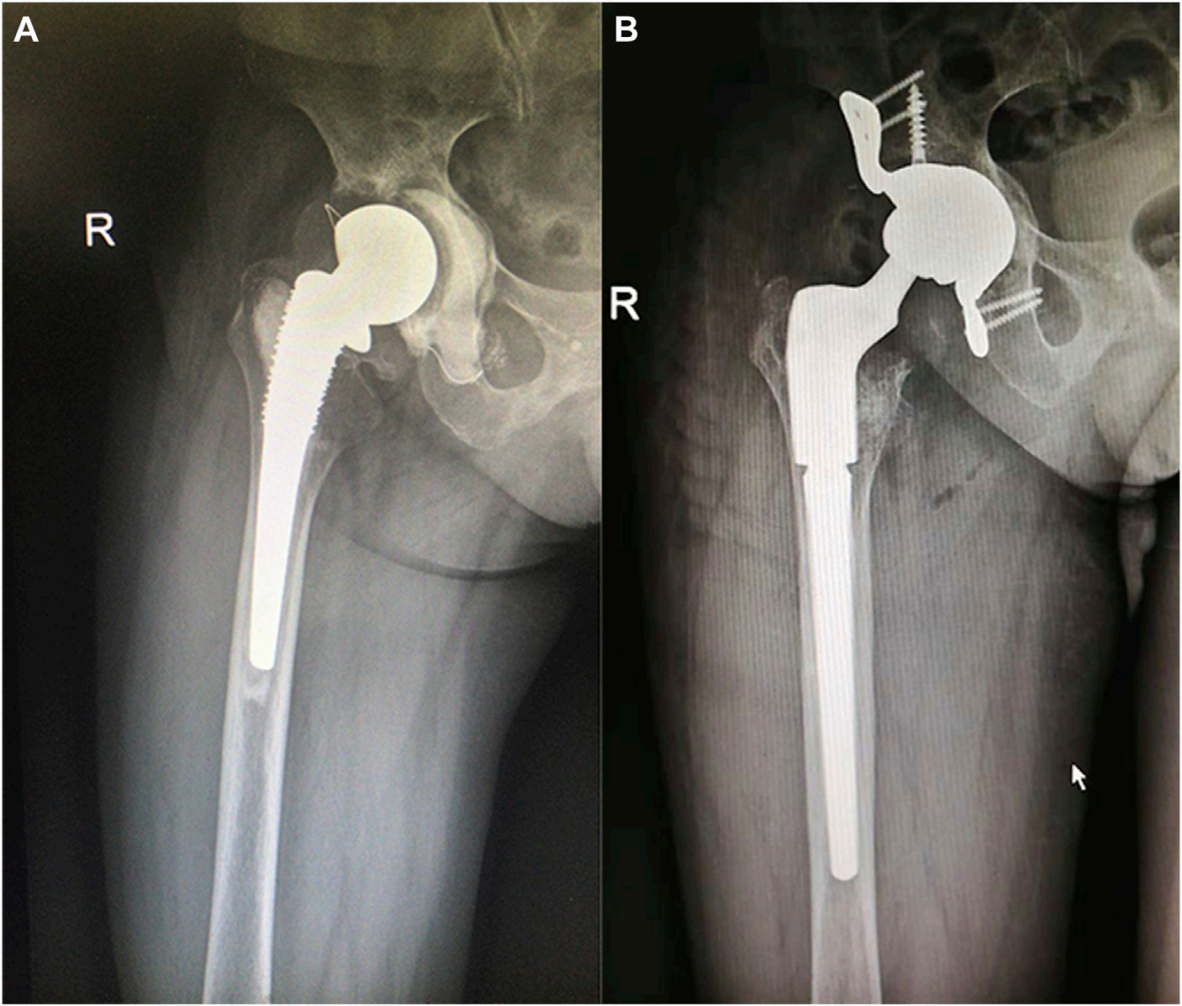
**(A)** A right total hip arthroplasty in a 62-year-old female patient, completed 16 years previously. The acetabulum side was supported with a cage. **(B)** Paprosky classification of the femur as being type 3A. An Arcos Modular Femoral Revision System stem was used with cancellous bone grafting of the proximal end.

**FIGURE 4:**
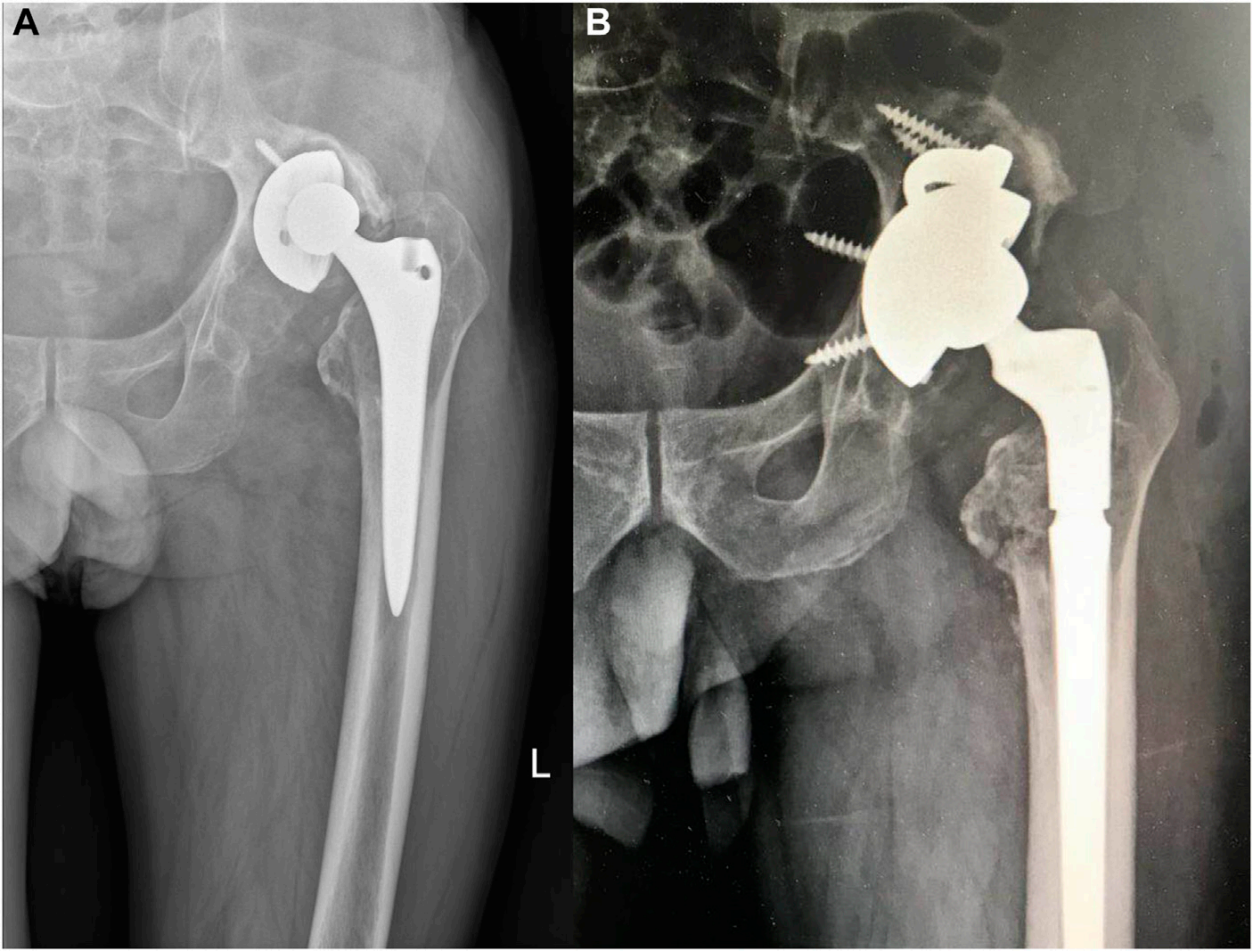
**(A)** A left total hip arthroplasty in a 66-year-old male patient, completed 10 years previously. The patient had experienced intermittent pain over a period of 3 years. **(B)** Paprosky classification of the femur as being type 3A.

Three patients underwent ETOs, and their bone had healed by the final follow-up. At the final follow-up, there were 21 bone ingrowth fixations and 10 stable fibrous fixations, and all stems were considered stable even though 10 stems had sunk more than 5 mm (6–20 mm). All morselized bone allografts had fused well with the host bones 12 months postoperatively, typical cases shown in Figures 3 and 4 in this text. At the final follow-up, three patients evidenced discontinuous translucent lines at the bone–prosthesis interface, mainly in Gruen 1, 2, and 6, with a maximum width of 2 mm, no progressive widening, and no 100% fine lines around the stem. According to the criteria set out in (Engh et al., 1990; Camilo et al., 2011), two cases showed slight stress shielding, one case showed moderate stress shielding, but no serious stress shielding occurred in any of the cases.

## Discussion

The combined ARCOS stem can be used for femoral revision after hip arthroplasty. No periprosthetic infection, prosthesis loosening, or prosthesis-sinking occurred 3 years after surgery, which can contribute to prolonging the length of the limb. Different degrees of bone defects may exist in the proximal femur among hip revision cases for various reasons. Dealing with lateral bone defects of the femur and associated deformity in the hip makes the revision procedure challenging. In the present study, the authors used a modular stem; the results indicated this to be an appropriate treatment for aseptic loosening revision.

In the current study, the ARCOS was used for patients with femoral bone defects beyond that of Paprosky type 2 but who still had good distal bone mass. In all cases, the prosthesis achieved satisfying initial clinical results. All prostheses had excellent initial stability without subsequent hip dislocation. Although there were eight cases of postoperative dislocation of the hip in the early stage of this study, the X-ray examination found that the prosthesis tilted forward well; the dislocation cases were considered to have been related to an incorrect postoperative posture. After manual reduction, a brace was fixed for 1 month. At the time of the final follow-up, no patient had experienced a dislocation. Picado et al. (2020) reported that the most common cause for reoperation was dislocation (3 hips, 7.3%, Restoration modular stem, Stryker Orthopedics). Sivananthan et al. (2017) reported on four stems that required revision because of infection, recurrent dislocation, or suboptimal implant position. Despite the high incidence of dislocation, there were no revisions due to dislocation in their study. Although specific studies posit no significant correlation between dislocation and prosthesis type, clinical dislocation has a strong relationship with femoral anteversion, particularly during the revision hip procedure, as the proximal femur structure is altered, making it challenging to reconstruct femoral anteversion. Thus, a modular design may assist a surgeon to make fewer errors regarding femoral anteversion (Alberton et al., 2002).

The modular ARCOS stem can adjust the length of the limb and reduce limb discrepancies. In our study, the postoperative limb was lengthened by 17.8 ± 9.8 mm (not including patients with Paprosky femoral defect type I). Feng et al. (2020) reported a preoperative leg length discrepancy of 18.7 ± 6.6 mm and a postoperative leg length discrepancy of 2.3 ± 2.7 mm in the use of modular stems (Link MP and AK-SL), including patients with Paprosky femoral defect types 1–3B. Regarding the leg length discrepancy, Camilo et al. (2011) reported that when using a modular stem for femoral revision, particularly for patients with Paprosky types 1 and 2 bone defects, the postoperative discrepancy of leg length was less than 5 mm. The modular stem effectively realized the predefined length of legs before surgery and avoided the problem of leg length discrepancy resulting from the use of non-modular stems (Desai et al., 2012). The ARCOS stems, compared with non-modular stems, provide flexibility for adjusting the leg length and offset during surgery. In addition, with modular stems, both metaphyseal and diaphyseal defects can be addressed independently. The current viewpoint is that the authors should put the acetabular prosthesis back into an anatomic position during surgery to restore the center of rotation.

While a modular stem presents advantages it also has risks. The main concern in this regard is breakage at the junction of its distal and proximal parts. Postoperative micro-motion and fatigue stress of the junction may lead to breakage between the distal and proximal parts of the prosthesis, failure of the locking mechanism, and dislodging of the proximate sleeve (Cross and Paprosky, 2013; Stimac et al., 2014). Lucena et al. (2020) reported that at a 5-year follow-up, there were no implant breakages and the survivorship was 100% free of the need for revision for aseptic loosening and 99% free of the need for revision for any reason concerning the femoral stem (Lucena et al., 2020). Sivananthan et al. (2017) reported no complications regarding modular junctions. The current study’s results were consistent with those of Lucena and Sivananthan. The ARCOS stem is reinforced with a patented roller reinforcement technology that triples the metal strength of the area in which it is placed, effectively reducing and avoiding breaking and microseismic wear of the joint. All ARCOS stems showed good fusion with the host bones 12 months postoperatively. During the surgery, morselized bone allografts were implanted in the bone defect to increase the stability and reduce the wear of the prosthesis (Fink, 2018).

## Limitations

This study had some limitations. First, the sample size was relatively small due to the small number of patients requiring revision, based on the aseptic loosening of a THA. In future studies, the authors aim to conduct multi-center trials with larger sample sizes to observe whether the results of this study can be confirmed. Second, the follow-up time was short, and the long-term use of the prosthesis could not be observed. No randomized controlled study was concurrently conducted; therefore, the use of the ARCOS stem could not be compared in this way. In addition, during the operation and follow-up process, patients’ treatment plans were determined only by the two doctors in charge. There may be have certain deviation, but the deviation error of the results can be minimized.

The modular taper stem is extremely versatile, can be used in most femoral revision cases, and allows for rapid bone reconstruction. However, the early dataset included in this study highlights the need for prolonging the follow-up study period to explore issues such as stress shielding around the prosthesis, the sinking of the prosthesis, and the mid and long-term prosthesis survival rate.

## Data Availability

The raw data supporting the conclusion of this article will be made available by the authors, without undue reservation.
